# Supplementary material for: A high throughput multi-locus insecticide resistance marker panel for tracking resistance emergence and spread in Anopheles gambiae
Source: Sci Rep. 2019 Sep 16;9:13335. doi: 10.1038/s41598-019-49892-6 (PMC6746726; doi:10.1038/s41598-019-49892-6)
Supplement: Supplementary file 1 — Supplementary figures and tables [file 41598_2019_49892_MOESM1_ESM.pdf]

A high throughput multi-locus insecticide resistance marker panel for tracking resistance emergence and spread in *Anopheles gambiae*

Eric R. Lucas, Kirk A. Rockett, Amy Lynd, John Essandoh, Nelson Grisales, Brigid Kemei, Harun Njoroge, Christina Hubbart, Emily J. Rippon, John Morgan, Arjen Van't Hof, Eric O. Ochomo, Dominic P. Kwiatkowski, David Weetman, Martin J. Donnelly

Electronic Supplementary Material

Supplementary figures and tables

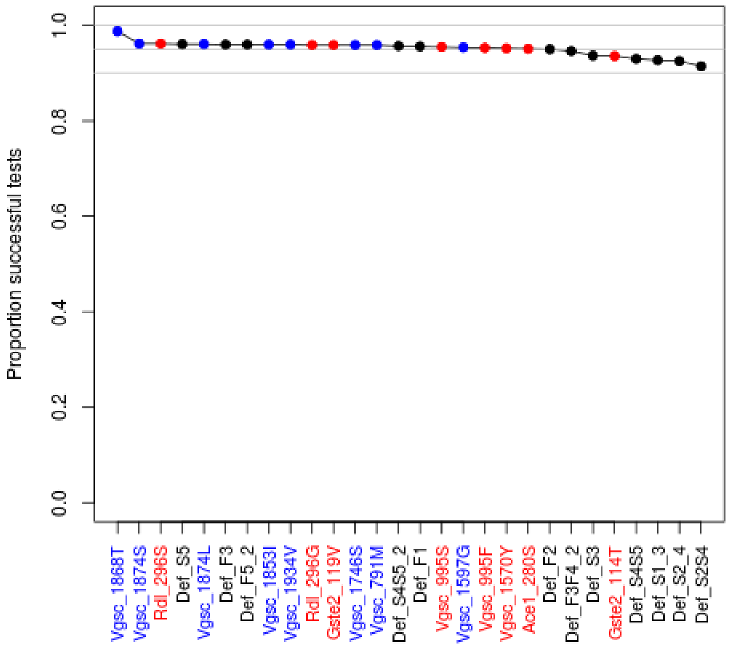

Fig. S1: Success rate of individual SNP assays in the multiplex sequenome assay was never lower than 90% (measured as the proportion of assays that were of high enough quality to give a genotype call). Red points indicate known resistance variants, blue points indicate variants found on the background of some *kdr* mutations and which may therefore be associated with either increased resistance or compensation for the costs of the *kdr* mutations, black points indicate SNPs used to distinguish between *kdr* mutant haplotype backgrounds.

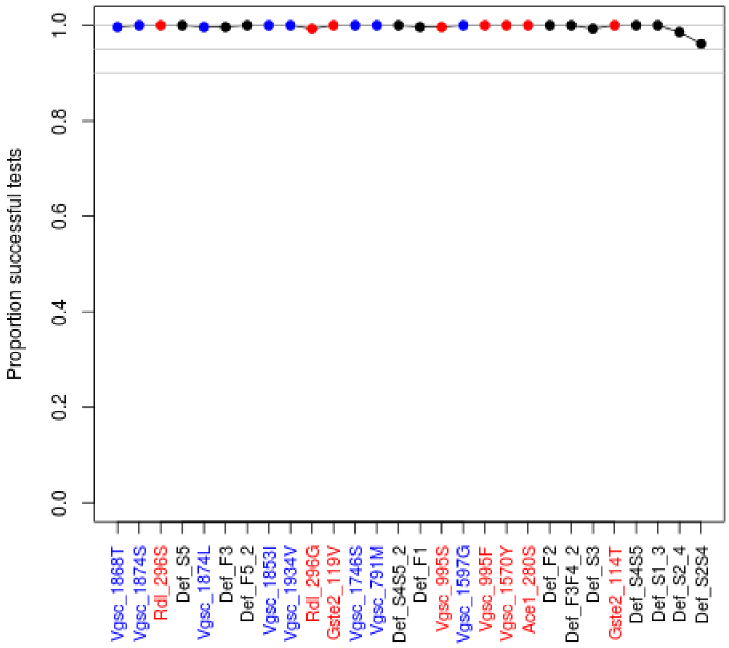

Fig. S2: Success rate of individual SNP assays among *An. coluzzii* samples was never lower than 95% (measured as the proportion of assays that were of high enough quality to give a genotype call). Red points indicate known resistance variants, blue points indicate variants found on the background of some *kdr* mutations, black points indicate SNPs used to distinguish between *kdr* mutant haplotype backgrounds.

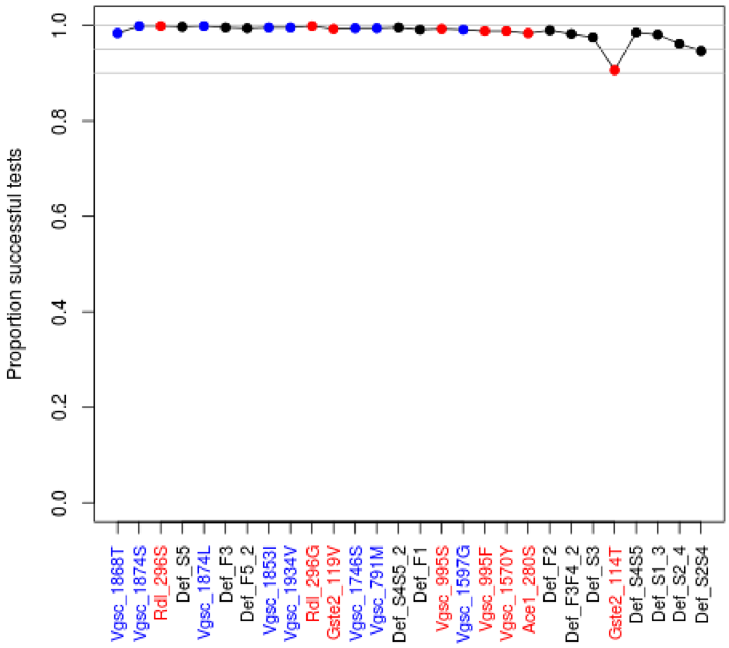

Fig. S3: Success rate of individual SNP assays among *An. gambiae* samples was never lower than 90% (measured as the proportion of assays that were of high enough quality to give a genotype call). Red points indicate known resistance variants, blue points indicate variants found on the background of some *kdr* mutations, black points indicate SNPs used to distinguish between *kdr* mutant haplotype backgrounds.

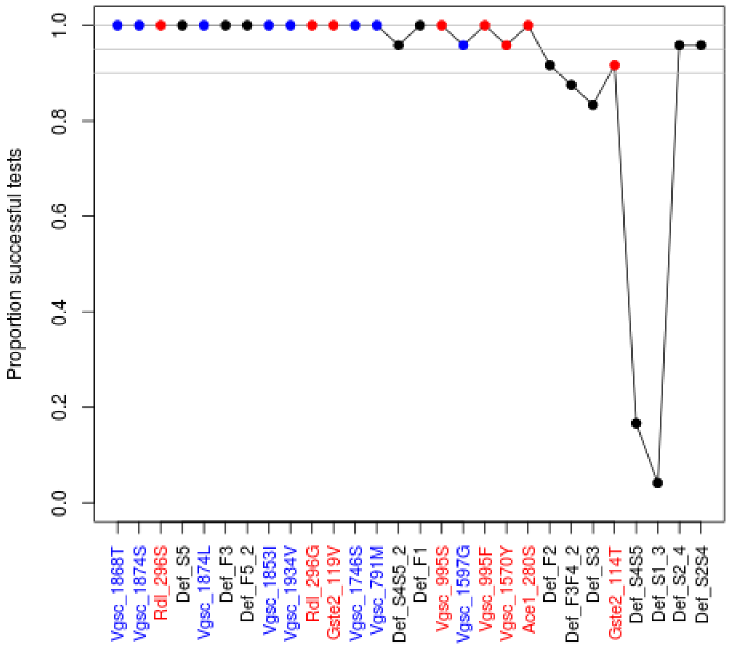

Fig. S4: Success rate of individual SNP assays among *An. arabiensis* samples was good overall, but some assays had a low rate of success (measured as the proportion of assays that were of high enough quality to give a genotype call). All four assays with success rates below 90% were ones used to distinguish between *kdr* haplotype backgrounds Red points indicate known resistance variants, blue points indicate variants found on the background of some *kdr* mutations, black points indicate SNPs used to distinguish between *kdr* mutant haplotype backgrounds.

Table **S1**: Bioassay results for samples from Burkina Faso (CDC bottle assay).

	20ppm Permethrin			
Location	Dead	Live	Total	Mortality
Bakaridjan	30	11	41	73.2%
Bounouba	15	26	41	36.6%
Naniagara	15	33	48	31.3%
Tiefora	24	52	76	31.6%

Table **S2**: Bioassay results for samples from DRC (WHO tube assay).

	0.75% Permethrin			
Location	Dead	Live	Total	Mortality
Pwamba	7	18	25	28.0%
Fiwa	98	83	181	54.1%
Bassa	101	45	146	69.2%

	0.05% Deltamethrin			
Location	Dead	Live	Total	Mortality
Pwamba	40	23	63	63.5%
Fiwa	131	44	175	74.9%
Bassa	119	24	143	83.2%

Table **S3**: Bioassay results for samples from Ghana (WHO tube assay).

	0.75% Permethrin			
Location	Dead	Live	Total	Mortality
Keta	61	50	111	55.0%

	0.05% Deltamethrin			
Location	Dead	Live	Total	Mortality
Keta	75	45	121	62.0%

	4% DDT			
Location	Dead	Live	Total	Mortality
Keta	43	66	110	39.1%

Table **S4**: Bioassay results for samples from Kenya WHO tube assay. Results are for *An. gambiae* s.l. in 2015.

	0.75% Permethrin			
Location	Dead	Live	Total	Mortality
Bondo	522	569	1091	47.8%
Nyando	319	198	517	61.7%
Rachuonyo	670	218	888	75.5%
Teso	332	409	741	44.8%

	0.05% Deltamethrin			
Location	Dead	Live	Total	Mortality
Bondo	1028	759	1787	57.6%
Nyando	537	342	879	61.1%
Rachuonyo	919	213	1132	81.2%
Teso	585	750	1335	43.8%

Table **S5**: SNPs VGC-1746S and VGC-791M are almost perfectly associated (only two samples give discordant calls). The wild-type alleles are G and C for 1746S and 791M respectively.

		VGC-1746S		
		GG	GT	TT
	CC	672	0	0
VGC-791M	CT	2	33	0
	TT	0	0	5
